# Predicting Successes and Failures of Clinical Trials With Outer Product–Based Convolutional Neural Network

**DOI:** 10.3389/fphar.2021.670670

**Published:** 2021-06-16

**Authors:** Sangwoo Seo, Youngmin Kim, Hyo-Jeong Han, Woo Chan Son, Zhen-Yu Hong, Insuk Sohn, Jooyong Shim, Changha Hwang

**Affiliations:** ^1^ Department of Data and Knowledge Service Engineering, Dankook University, Gyeonggido, Korea; ^2^ Department of Statistics, Dankook University, Gyeonggido, Korea; ^3^ Department of Pathology, College of Medicine, University of Ulsan, Asan Medical Center, Seoul, Korea; ^4^ Arontier, Seoul, Korea; ^5^ Department of Statistics, Institute of Statistical Information, Inje University, Gyeongsangnamdo, Korea

**Keywords:** clinical trial, convolutional neural network, multimodal learning, outer product, imbalance

## Abstract

Despite several improvements in the drug development pipeline over the past decade, drug failures due to unexpected adverse effects have rapidly increased at all stages of clinical trials. To improve the success rate of clinical trials, it is necessary to identify potential loser drug candidates that may fail at clinical trials. Therefore, we need to develop reliable models for predicting the outcomes of clinical trials of drug candidates, which have the potential to guide the drug discovery process. In this study, we propose an outer product–based convolutional neural network (OPCNN) model which integrates effectively chemical features of drugs and target-based features. The validation results via 10-fold cross-validations on the dataset used for a data-driven approach PrOCTOR proved that our OPCNN model performs quite well in terms of accuracy, F1-score, Matthews correlation coefficient (MCC), precision, recall, area under the curve (AUC) of the receiver operating characteristic, and area under the precision–recall curve (AUPRC). In particular, the proposed OPCNN model showed the best performance in terms of MCC, which is widely used in biomedicine as a performance metric and is a more reliable statistical measure. Through 10-fold cross-validation experiments, the accuracy of the OPCNN model is as high as 0.9758, F1 score is as high as 0.9868, the MCC reaches 0.8451, the precision is as high as 0.9889, the recall is as high as 0.9893, the AUC is as high as 0.9824, and the AUPRC is as high as 0.9979. The results proved that our OPCNN model shows significantly good prediction performance on outcomes of clinical trials and it can be quite helpful in early drug discovery.

## Introduction

Over the past 30 years, failures at all phases of clinical trials have increased rapidly for safety reasons (Ledford, 2011; Hay et al., 2014; Lysenko et al., 2018; Liu et al., 2021). This phenomenon happens despite significant improvements at all stages of the drug development pipeline (Scannell et al., 2012). There have been many improvements in screening for drugs that are likely to fail clinical trials.

Drug-likeness scores are widely utilized as a useful guideline for eliminating toxic molecules during the early stages of drug development. This concept was first introduced by Lipinski’s rule of five (Ro5), which screens molecules with a low probability of useful oral activity due to poor absorption or permeation (Lipinski et al., 1997). That is to say, the Ro5 enhanced the drug discovery process because it helps in distinguishing between drug-like and nondrug-like molecules. However, Lipinski argued that the Ro5 is a very conservative strategy because this rule does not guarantee drug-likeness (Lipinski, 2004). To enhance the Ro5, Veber’s rule and Ghose’s rule were proposed (Ghose et al., 1999; Veber et al., 2002). The quantitative estimate for drug-likeness (QED) was also recently proposed as an alternative to rule-based methods (Bickerton et al., 2012).

Despite lots of advances in identifying potentially toxic drugs, overall failure rates of clinical trials continued to increase (Hay et al., 2014). To deal with this problem, Gayvert et al. recently proposed a new data-driven approach PrOCTOR, which predicts the odds of clinical trial outcomes on the basis of random forests that integrates chemical properties of drugs and target-based properties (Gayvert et al., 2016). It was exhibited that both the chemical features and target-related gene expression values contribute to effective classification. In this study, we will also use the chemical features of drugs and target-based features for predicting successes and failures of clinical trials. Lo et al. applied machine learning techniques to predict the outcomes of randomized clinical trials using drug development and clinical trial data (Lo et al., 2019). Munos et al. improved the prediction of clinical success using machine learning algorithms based on a large database of projects (Munos et al., 2020).

Modeling the relationship between chemical structure of drug and molecular activity is very important for drug development for precision medicine. In this study, we employ a novel outer product–based convolutional neural network (OPCNN) to integrate effectively chemical features of the drugs, biological network features, genotype-tissue expression (GTEx) features, and target loss frequency. The purpose of this research is to propose a two-dimensional (2D) convolutional neural network (CNN) based on the outer product of chemical feature vector and a target-based feature vector to predict successes and failures of clinical trials.

## Materials and Methods

### Dataset

We evaluated our proposed OPCNN using the same dataset as in Gayvert et al. (Gayvert et al., 2016), which consists of 757 approved drugs for positive class and 71 failed drugs for negative class. We notice that the dataset is imbalanced. The imbalance ratio of majority to minority compounds is 10.662. The set of 47 input features describing each drug contains 10 molecular properties, 34 target-based properties, and three drug-likeness rule outcomes for the Lipinski’s rule of five, Veber’s, and Ghose’s rules. There are several missing values for six features. We impute them with relevant median values. Molecular properties represent molecular weight, XLogP, polar surface area, hydrogen bond donor and acceptor counts, formal charge, number of rings, rotatable bond count, refractivity, and logP solubility. For a set of 30 target-based features, we use the median expression of each drug’s known gene targets in 30 different tissues, including the blood, skin, brain, liver, testis, muscle, nerve, and heart, calculated from the GTEx project. For three other target-based features, we use the network connectivity of the target, with the gene degree feature and betweenness feature computed using an aggregated gene–gene interaction network. We also use a feature that represents the loss-of-function mutation frequency in the target gene.

### Model Development

#### The Proposed OPCNN Classifier

The problem of predicting clinical successes and failures of clinical trials is modeled as a binary classification task. For a given drug i, the target label is a binary variable $y_{i}$, where $y_{i}=1$ indicates that the drug is passed and $y_{i}=0$ indicates otherwise. Our dataset contains n=828 drugs, where each is represented by a pair of feature vector $x_{i}$ and a corresponding clinical outcome $y_{i}$: ${\left(x_{i},\;\;y_{i}\right)}_{i=1}^{n}$, where $x_{i}=\left(x_{i}^{\left(1\right)},x_{i}^{\left(2\right)}\right)$ and $x_{i}^{\left(1\right)}$ and $x_{i}^{\left(2\right)}$ represent the chemical feature vector and target-based feature vector, respectively. The data associated with this task are bimodal and highly imbalanced. Both modalities are associated with chemical properties of the drugs and target-based properties, respectively. Thus, we need to join effectively two different modalities. In addition, we also need to consider the model that deals with class-imbalance problem.

**FIGURE 1:**
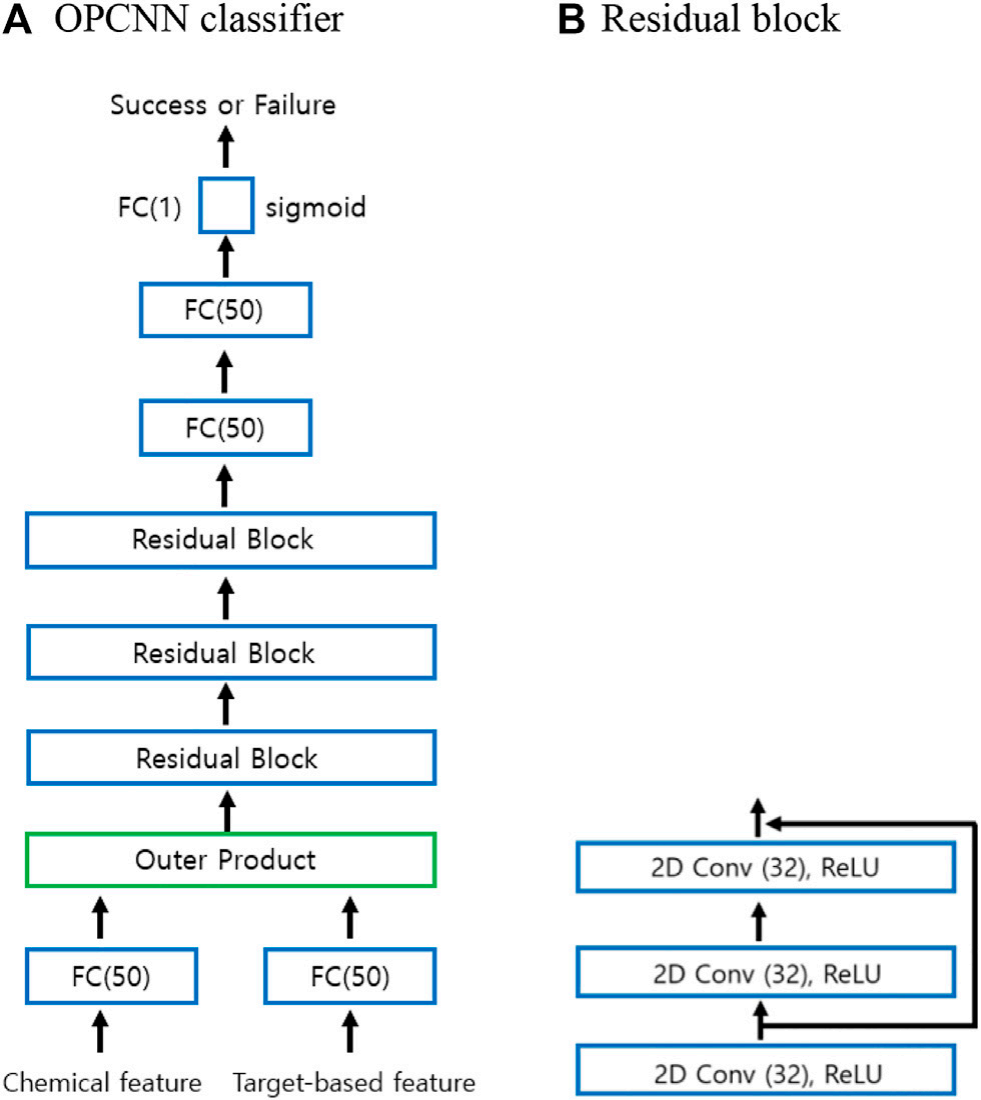
A workflow of the proposed OPCNN classifier for predicting successes and failures of clinical trials. Given an outer product of two representative feature vectors as an input, 2D CNN is used to learn features. The architecture of OPCNN consists of three residual blocks and five fully connected (FC) layers. Each residual block has three convolution layers. **(A)** OPCNN classifier **(B)** Residual block.

Figure 1 explains the entire workflow of the proposed OPCNN classifier for the prediction of successes and failures in clinical trials. Our OPCNN consists of three residual blocks and five fully connected (FC) layers. Each residual block has three convolution layers, each of which employs 32 kernels with kernel size 3 and stride size 1, and the rectified linear unit (ReLU) activation function. The numbers in parentheses of FC(1), FC(50), and FC(100) indicate the number of nodes. FC(1) layer employs the sigmoid activation function. Both FC(50) and FC(100) layers employ the rectified linear unit (ReLU) activation function. Our method consists of two stages. First, the representative feature vectors of chemical feature vector and target-based feature vector are calculated and then the outer products between these two representative feature vectors are calculated. Second, a 2D CNN model is adopted to extract deep features from the outer products and to predict successes and failures of clinical trials.

The process of calculating the outer product is as follows. The chemical feature vector $x^{\left(1\right)}\in R^{13}$ and the target-based feature vector $x^{\left(2\right)}\in R^{34}$ in different modalities are first fed into the FC(50) layer to get representative feature vectors $f^{\left(1\right)}\in R^{50}$and $f^{\left(2\right)}\in R^{50}$ and improve their performance. Given $f^{\left(1\right)}\in R^{50}$ and $f^{\left(2\right)}\in R^{50}$, the outer product on the augmented unimodal is calculated as follows:

$$x^{f}=\left[\begin{array}{l} f^{\left(1\right)}\\ 1 \end{array}\right]⊗\left[\begin{array}{l} f^{\left(2\right)}\\ 1 \end{array}\right]=\left[\begin{array}{ll} f^{\left(1\right)} & f^{\left(1\right)}⊗f^{\left(2\right)}\\ 1 & f^{\left(2\right)t} \end{array}\right].$$ (1)

Here, ⊗ indicates the outer product between vectors. Thus, this outer product produces two sets of information: the bimodal interactions in the form of two-dimensional tensor and the raw unimodal representations of the modalities. The tensor calculated by such outer product is directly fed into the first residual block. The final representation is used for the classification task.

#### Other Deep Multimodal Neural Networks

Classification with multimodal data often occurs in many machine learning applications (Baltrušaitis et al., 2019; Gao et al., 2020). Multimodal learning is an effective approach to combine information from multiple modalities to perform a prediction task. The modalities may be independent or correlated. Fusing multiple modalities is a key issue in any multimodal task. In general, the fusion of multiple modalities can be achieved at three levels: at the level of features or at a lower layer, at the intermediate level, and at the level of decisions. Fusion at the feature level or at a lower layer is called early fusion. On the other hand, fusion at the intermediate layer is called intermediate fusion, whereas fusion at the level of decisions is called late fusion. Because early and late fusions can generally suppress either intra-modality or inter-modality interactions, recent studies have focused on intermediate methods that allow fusion to occur on multiple layers of a deep model.

**FIGURE 2:**
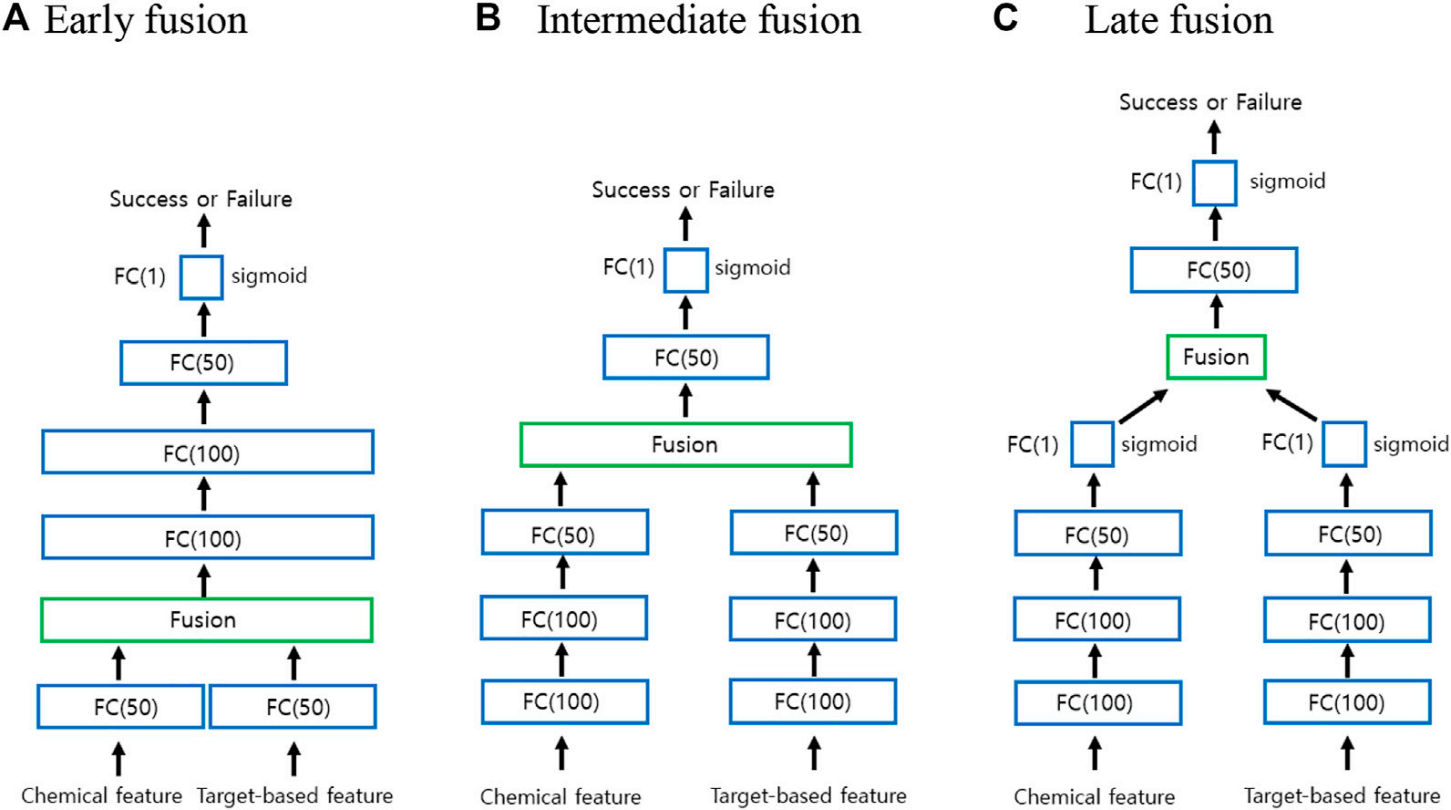
Graphical representation for the early, intermediate, and late fusions. **(A)** Early fusion **(B)** Intermediate fusion **(C)** Late fusion.

Figure 2 illustrates a graphical representation for deep multimodal neural network (DMNN) models associated with the early, intermediate, and late fusions used in the study. As seen from Figure 2, each DMNN model consists of several FC layers. The number in parentheses indicates the number of nodes. As in Figure 1, the FC(1) layer employs the sigmoid activation function. Both FC(50) and FC(100) layers employ the ReLU activation function. In the case of early fusion, each modality is first fed into an FC(50) layer before fusion in order to improve performance and to apply several fusion techniques. However, the standard early fusion allows multiple modalities to be directly concatenated to produce a single multimodal vector. In the case of intermediate and late fusions, each modality is fed into an independent deep neural network (DNN) and then fused to be the inputs of higher layers. The final representation is used for the classification task.

Based on the literature, five fusion operations are often used to fuse multiple modalities (Feng et al., 2021): Eq. 1 addition, Eq. 2 product, Eq. 3 concatenation, Eq. 4 ensemble, and Eq. 5 mixture of experts. Addition and product operations are performed in terms of elements at the fusion layer. Here, we will consider two more multimodal fusion techniques based on tensor fusion layer (TFL) (Zadeh et al., 2017) and multimodal circulant fusion (MCF) (Wu and Han, 2018) for early and intermediate fusions. When using TFL and MCF for the intermediate fusion, we actually use the DMNN model with FC(100)-FC(50) instead of FC(100)-FC(100)-FC(50) for each modality to improve its performance.

In general, the early fusion approach performs better than individual unimodal classifiers. The ensemble approach called late fusion is to weigh several individual classifiers and combine them to get a classifier that surpasses individual classifiers. In general, ensemble methods provide better results when there are significant differences among the models. Therefore, many ensemble methods try to enhance diversity among the models to be combined. Based on our preliminary studies, the unimodal classifiers using only chemical features perform better than unimodal classifiers using only target-based features. We actually have tried three different ensemble models using support vector machine (SVM) (Vapnik, 1995) and one-dimensional CNN and our DMNN for the late fusion in Figure 2. Note that our DMNN model uses only concatenation technique for late fusion. Since our DMNN ensemble model has shown the best performance, we will only report those results later.

#### Tensor Fusion Layer and Multimodal Circulant Fusion

We now briefly illustrate TFL and MCF strategies. Element-wise addition and product are used to join features from multiple modalities. Concatenation technique focuses more on learning intra-modality than learning inter-modality. However, both TFL and MCF capture both intra-modality and inter-modality dynamics. TFL also employs the same outer product on the augmented unimodal as in our OPCNN.

We first illustrate the idea of TFL strategy to fuse multimodal data at the tensor level. For our studies, we need to build a TFL that disentangles unimodal and bimodal dynamics. Given representative feature vectors $f^{\left(1\right)}\in R^{50}$ and $f^{\left(2\right)}\in R^{50}$ associated with the chemical feature vector $x^{\left(1\right)}\in R^{13}$ and the target-based feature vector $x^{\left(2\right)}\in R^{34}$ in different modalities, TFL calculates the outer product on the augmented unimodal using the Eq. 1. However, as seen from Figure 2, $f^{\left(1\right)}\in R^{50}$ and $f^{\left(2\right)}\in R^{50}$ are obtained slightly differently for the early fusion and intermediate fusion. Thus, TFL also produces two sets of information: the bimodal interactions in the form of two-dimensional tensor and the raw unimodal representations of the modalities. The tensor calculated by TFL is fed into a FC layer after being flattened. It is noted that TFL introduces no learnable parameters. Although TFL yields the high dimensional output tensor, chances of overfitting are low (Zadeh et al., 2017).

We now briefly illustrate the idea of MCF strategy which consists of four steps. Given representative feature vectors $f^{\left(1\right)}\in R^{50}$ and $f^{\left(2\right)}\in R^{50}$, we first project $f^{\left(1\right)}$ and $f^{\left(2\right)}$ to a lower dimensional space using projection matrices $W_{1}\in R^{d\times 50}$ and $W_{2}\in R^{d\times 50}$.

$$v=W_{1}f^{\left(1\right)}\in R^{d}and\;c=W_{2}f^{\left(2\right)}\in R^{d},$$ (2)

where d≤50. As in TFL, $f^{\left(1\right)}\in R^{50}$ and are obtained slightly differently for early fusion and intermediate fusion. Second, we construct circulant matrices $A\in R^{d\times d}$ and $B\in R^{d\times d}$ using the projection vector $v\in R^{d}$ and $c\in R^{d}$.

A=circ(v), B=circ(c), (3)

where circ(b) denotes converting b to a circulant matrix. Third, we calculate in one of two ways: matrix multiplication between circulant matrix and projection vector to make elements in this matrix and vector fully interact. Two ways are illustrated in Eqs. 4, 5.

f=Ac, g=Bv , (4)

$$f=\frac{1}{d}\overset{d}{\underset{i=1}{\sum }}a_{i}⊙c,\;g=\frac{1}{d}\overset{d}{\underset{i=1}{\sum }}b_{i}⊙v.$$ (5)

Here, $a_{i}$ and $b_{i}$ are column vectors of circulant matrices A and B, respectively. ⊙ denotes the operation of element-wise product. It is noted that we introduce no new parameters in the multiplication operation. Finally, we calculate target vector $m\in R^{k}$ using f, g, and a projection matrix $W_{3}\in R^{k\times d}$.

$$m=W_{3}\left(f⊕g\right)\in R^{k}.$$ (6)

Here, ⊕ denotes the operation of element-wise addition.

#### Imbalanced Data Learning

Since the ratio of passed drugs to failed drugs in clinical trials is highly imbalanced, the class-imbalance problem occurs. There are generally three types of methods to deal with the imbalance data learning (Wang et al., 2019). We briefly illustrate the methods to be actually used in the study. 1) Sampling method: an intuitive way to cope with the imbalanced distribution of the data is to balance class distributions via resampling, which could oversample the minority class and undersample the majority class. One advanced sampling method called synthetic minority oversampling technique (SMOTE) creates artificial examples through interpolating neighboring data points (Chawla et al., 2002). Several variants of this technique have been proposed. However, oversampling can lead to overfitting due to repeatedly visiting the existing minority samples. On the other hand, undersampling can discard potentially useful information in majority samples. 2) Cost-sensitive learning method: instead of balancing class distributions via sampling methods, this method aims at coping with the abovementioned issues by directly imposing a heavier cost on misclassifying the minority class. However, what types of cost to use in different problem settings is still an open problem. In this study, we use the cost-sensitive learning method using the class weights (CWs) $n/\left(2\times n_{+}\right)$ and $n/\left(2\times n_{-}\right)$ for the positive and negative classes, respectively. Recall that the majority class is the positive class and the minority class is the negative class in the study. Here, n represents the size of training dataset and $n_{+}$ and $n_{-}$ represent the sizes of the positive and negative classes, respectively. 3) Hybrid method: this is an approach that combines aforementioned two methods. In the study, we use the combination of SMOTE and CW techniques.

### Classification Evaluation Metrics

To evaluate binary classifications, we can employ various statistical metrics, accordingly to the goal of the experiment we are performing. Accuracy and F1-score have been among the most quintessential metrics for binary classification problems. Accuracy is a valid evaluation metric for classification problems which are well balanced and not skewed or no class imbalance. In general, accuracy can dangerously show overoptimistic inflated results, especially on imbalanced datasets. F1-score is the harmonic mean of precision and recall, and thus F1-score maintains a balance between the precision and recall for classifier. F1-score is a measure of accuracy, which takes both false positives and false negatives into account. F1-score is usually more useful than accuracy especially for imbalanced classification. Precision and recall are two extremely important model evaluation metrics. While precision measures the probability of correct detection of positive values, recall measures the ability to distinguish between the classes. Area under the curve (AUC) of the receiver operating characteristic (ROC) and the area under the precision–recall curve (AUPRC) are ranking order metrics. AUPRC is often used as evaluation metrics for imbalanced classes. AUPRC is preferred over AUC. When comparing performance of classifiers that need to deal with imbalanced data, F1-score, precision–recall, and AUPRC are often used out of convenience (Brabec et al., 2020).

The use of inadequate performance metrics, such as accuracy, lead to poor generalization results because the classifiers tend to predict the largest size class. Matthews correlation coefficient (MCC) is widely used in biomedicine as a performance metric. The MCC is a more reliable statistical measure which produces a high score only if the prediction obtained good results in all of the four confusion matrix categories (true positives, false negatives, true negatives, and false positives), proportional to both the size of positive elements and the size of negative elements in the dataset (Chicco and Jurman, 2020; Ietswaart et al., 2020). MCC is easier to interpret as a correlation coefficient since it takes a value in the interval [−1, 1], with 1 showing a perfect classifier, –1 showing a perverse classifier, and 0 showing that the prediction is uncorrelated with the ground truth. MCC is a very good metric for the imbalanced classification and can be safely used for even classes that are very different in sizes. It is also shown that MCC produces a more informative and truthful score in evaluating binary classifications than accuracy and F1-score (Chicco and Jurman, 2020). We prefer to use MCC to assess classification performance in this study.

The performance of the prediction models of successes and failures of clinical trials is evaluated using the following statistical metrics: TN (true negative), FN (false negative), TP (true positive), FP (false positive), PR (precision), RE (recall), ACC (accuracy), F1-score, MCC, AUC, and AUPRC, which are defined in the following equations:

$$PR=\frac{TP}{TP+FP},$$ (7)

$$RE=\frac{TP}{TP+FN},$$ (8)

$$ACC=\frac{TP+TN}{TP+FN+FP+TN},$$ (9)

$$F1-score=2\times \frac{PR\times RE}{PR+RE},and$$ (10)

$$MCC=\frac{TP\times TN-FP\times FN}{\sqrt{\left(TP+FN\right)\left(TP+FP\right)\left(TN+FP\right)\left(TN+FN\right)}}.$$ (11)

## Experiments and Results

As mentioned before, we use the same dataset as in Gayvert et al. (Gayvert et al., 2016), which consists of 757 passed drugs for positive class and 71 failed drugs for negative class. We notice that the dataset is imbalanced. The imbalance ratio of majority to minority compounds is 10.662. The dataset used may not have enough samples for the use of deep learning. We use 10-fold cross-validation techniques to evaluate classification models. The folds are stratified based on drugs. That is to say, all experiments of a single drug are either completely in the training set or completely in the test set. Thus, a model is expected to predict the clinical outcomes of previously unseen drugs at test time. We conduct these 10-fold cross-validation experiments, randomly splitting ten folds. To obtain reliable performance results, we repeat the cross-validation 20 times for each model on the dataset, and report the mean and standard deviation for each metric.

We select OPCNN as a good model for this particular data. Early experiments with different models did not yield meaningful results. To take into account the class imbalance, we use cost-sensitive learning and hybrid methods. We use binary cross entropy (BCE) as the loss function. We investigate the effect of employing weighted BCE and SMOTE to address the imbalance in our training dataset. Adam optimizer is used for training the neural networks. While the learning rate for Adam optimizer is tuned separately for each model and dataset pair, the same set of hyperparameters is used across the folds. We select hyperparameters such as the number of layers and the number of nodes for OPCNN and DMNN, which provide the best MCC value based on a 10-fold cross-validation.

Deep learning models are likely to overfit the training data since the data used do not have sufficient samples. Therefore, we consider two conventional machine learning models such as SVM and random forest for comparison since these models alleviate overfitting by ensemble and regularization techniques, respectively. 47 input features are first concatenated to be used as inputs of these two models. For the case of SVM, the polynomial kernel of degree 3 and penalty constant C=10 are selected. It is because this combination provides the best MCC value based on a 10-fold cross-validation. We have tried with several polynomial degrees and C values to determine the best combination. We have also tried with several kernel parameter values of Gaussian kernel and C values. For the case of random forest, the number of trees is selected as 100, which provides the best MCC value based on 10-fold cross validation. We have decided it by increasing the number of trees from 10 to 150 in increments of 10. When looking for the best split, the number of input features to be considered is determined as $\sqrt{47}$, where the number of input features is 47.

To statistically evaluate the significant improvement of our OPCNN, we utilize the two sided t-test. We basically compare the model with the best performance result to other models. For all evaluation metrics, the value for the best-performing model is highlighted in bold font. Therefore, the null hypotheses associated with Table 1 are given as follows: $H_{0}:ACC\left(\text{best model}\right)=ACC\left(\text{other model}\right)$, $H_{0}:F1-score\left(\text{best model}\right)=F1-score\left(\text{other model}\right),$ $H_{0}:MCC\left(\text{best model}\right)=MCC\left(\text{other model}\right)$, $H_{0}:Precision\left(\text{best model}\right)=Precision\left(\text{other model}\right),$ $H_{0}:Recall\left(\text{best model}\right)=Recall\left(\text{other model}\right)$, $H_{0}:AUC\left(\text{best model}\right)=AUC\left(\text{other model}\right)S$, and $H_{0}:AUPRC\left(\text{best model}\right)=AUPRC\left(\text{other model}\right)$. As seen from Table 1, the best model is OPCNN base model for the other five metrics except precision and recall. The relevant p-values less than 0.05 are given one asterisk, p-values less than 0.01 are given two asterisks, and p-values less than 0.001 are given three asterisks.

**TABLE 1:** Classification results for various prediction models via a 10-fold cross-validation.

Multimodal learning	Model	ACC	F1-score	MCC	Precision	Recall	AUC	AUPRC
SVM Early fusion Concatenation	Base	0.8308^***^ (0.0053)	0.9055^***^ (0.0031)	0.1796^***^ (0.014)	0.9253^***^ (0.0017)	0.8864^***^ (0.0054)	0.5622^***^ (0.0099)	0.9578^***^ (0.0099)
	CW	0.8152^***^ (0.0075)	0.8956^***^ (0.0046)	0.1837^***^ (0.0149)	0.9263^***^ (0.002)	0.8669^***^ (0.0079)	0.5658^***^ (0.0114)	0.9574^***^ (0.0011)
	SMOTE + CW	0.7748^***^ (0.0077)	0.8687^***^ (0.005)	0.1975^***^ (0.0157)	0.9297^***^ (0.0028)	0.8153^***^ (0.0085)	0.5791^***^ (0.0145)	0.9569^***^ (0.0015)
Random forest Early fusion Concatenation	Base	0.9149^***^ (0.0018)	0.9551^***^ (0.0009)	0.2018^***^ (0.0303)	0.9206^***^ (0.0013)	0.9924^***^ (0.0016)	0.7019^***^ (0.0058)	0.9532^***^ (0.0015)
	CW	0.9156^***^ (0.0022)	0.9556^***^ (0.0011)	0.1865^***^ (0.0367)	0.9193^***^ (0.0012)	**0.9950** (0.0018)	0.7125^***^ (0.0055)	0.9565^***^ (0.0021)
	SMOTE + CW	0.8949^***^ (0.0026)	0.9435^***^ (0.0014)	0.2484^***^ (0.0156)	0.9285^***^ (0.0012)	0.9689^***^ (0.0023)	0.7045^***^ (0.0052)	0.9577^***^ (0.0015)
OPCNN	Base	**0.9758** (0.0067)	**0.9868** (0.0037)	**0.8451** (0.0424)	0.9844^***^ (0.0050)	0.9893^***^ (0.0058)	**0.9824** (0.0102)	**0.9979** (0.0015)
	CW	0.9539^***^ (0.0249)	0.9743^***^ (0.0144)	0.7620^***^ (0.0854)	0.9866^**^ (0.0041)	0.9628^***^ (0.0282)	0.9653^***^ (0.0247)	0.9952^***^ (0.0045)
	SMOTE + CW	0.9329^***^ (0.0338)	0.9619^***^ (0.0201)	0.7012^***^ (0.0909)	0.9889 (0.0048)	0.9373^***^ (0.0379)	0.9583^***^ (0.0177)	0.9583^***^ (0.0177)
DMNN Early fusion Addition	Base	0.9653^***^ (0.0038)	0.9811^***^ (0.0021)	0.7727^***^ (0.0250)	0.9760^***^ (0.0037)	0.9863^***^ (0.0041)	0.9717^***^ (0.0061)	0.9968^**^ (0.0010)
	CW	0.9492^***^ (0.0075)	0.9719^***^ (0.0042)	0.7238^***^ (0.0368)	0.9843^***^ (0.0039)	0.9598^***^ (0.0065)	0.9660^***^ (0.0080)	0.9961^**^ (0.0011)
	SMOTE + CW	0.9309^***^ (0.0073)	0.9612^***^ (0.0043)	0.6740^***^ (0.0228)	0.9871^**^ (0.0032)	0.9367^***^ (0.0089)	0.9551^***^ (0.0070)	0.9944^***^ (0.0011)
DMNN Early fusion Product	Base	0.9669^***^ (0.0026)	0.9819^***^ (0.0014)	0.7880^***^ (0.0175)	0.9798^***^ (0.0030)	0.9840^***^ (0.0031)	0.9748^**^ (0.0055)	0.9972 (0.0012)
	CW	0.9449^***^ (0.0085)	0.9694^***^ (0.0048)<	0.7111^***^ (0.0358)	0.9849^***^ (0.0028)	0.9544^***^ (0.0080)	0.9678^***^ (0.0073)	0.9964^**^ (0.0011)
	SMOTE + CW	0.9170^***^ (0.0090)	0.9529^***^ (0.0054)	0.6465^***^ (0.0265)	0.9898 (0.0024)	0.9187^***^ (0.0095)	0.9560^***^ (0.0108)	0.9936^***^ (0.0061)
DMNN Early fusion Concatenation	Base	0.9652^***^ (0.0038)	0.9810^***^ (0.0021)	0.7715^***^ (0.0261)	0.9761^***^ (0.0035)	0.9861^***^ (0.0031)	0.9751^***^ (0.0063)	0.9973 (0.0008)
	CW	0.9473^***^ (0.0057)	0.9708^***^ (0.0032)	0.7125^***^ (0.0293)	0.9831^***^ (0.0031)	0.9589^***^ (0.0043)	0.9662^***^ (0.0071)	0.9963^**^ (0.0010)
	SMOTE + CW	0.9345^***^ (0.0069)	0.9634^***^ (0.0039)	0.6786^***^ (0.0301)	0.9855^***^ (0.0036)	0.9422^***^ (0.0064)	0.9569^***^ (0.0097)	0.9943^***^ (0.0019)
DMNN Early fusion TFL	Base	0.9652^***^ (0.0053)	0.9811^***^ (0.0029)	0.7700^***^ (0.0365)	0.9753^***^ (0.0039)	0.9869^***^ (0.0031)	0.9748^**^ (0.0064)	0.9971^*^ (0.0009)
	CW	0.9512^***^ (0.0072)	0.9731^***^ (0.0041)	0.7252^***^ (0.0324)	0.9822^***^ (0.0028)	0.9641^***^ (0.0070)	0.9663^***^ (0.0072)	0.9963^**^ (0.0009)
	SMOTE + CW	0.9172^***^ (0.0113)	0.9531^***^ (0.0067)	0.6387^***^ (0.0295)	0.9874^*^ (0.0032)	0.9212^***^ (0.0128)	0.9535^***^ (0.0085)	0.9943^***^ (0.0015)
DMNN Early fusion MCF	Base	0.9582^***^ (0.0048)	0.9773^***^ (0.0026)	0.7219^***^ (0.0309)	0.9703^***^ (0.0028)	0.9845^***^ (0.0041)	0.9635^***^ (0.0091)	0.9959^***^ (0.0016)
	CW	0.9325^***^ (0.0133)	0.9625^***^ (0.0076)	0.6429^***^ (0.0512)	0.9768^***^ (0.0040)	0.9487^***^ (0.0131)	0.9454^***^ (0.0131)	0.9939^***^ (0.0017)
	SMOTE + CW	0.8958^***^ (0.0122)	0.9407^***^ (0.0073)	0.5616^***^ (0.0345)	0.9798^***^ (0.0037)	0.9046^***^ (0.0126)	0.9331^***^ (0.0105)	0.9919^***^ (0.0021)
DMNN Intermediate fusion Addition	Base	0.9582^***^ (0.0041)	0.9773^***^ (0.0023)	0.7212^***^ (0.0261)	0.9701^***^ (0.0033)	0.9845^***^ (0.0041)	0.9630^***^ (0.0065)	0.9959^***^ (0.0008)
	CW	0.9315^***^ (0.0075)	0.9620^***^ (0.0043)	0.6335^***^ (0.0298)	0.9757^***^ (0.0036)	0.9487^***^ (0.0083)	0.9429^***^ (0.0090)	0.9934^***^ (0.0014)
	SMOTE + CW	0.9297^***^ (0.0081)	0.9607^***^ (0.0047)	0.6522^***^ (0.0291)	0.9820^***^ (0.0029)	0.9404^***^ (0.0083)	0.9462^***^ (0.0065)	0.9934^***^ (0.0010)
DMNN Intermediate fusion Product	Base	0.9484^***^ (0.0047)	0.9716^***^ (0.0026)	0.6983^***^ (0.0265)	0.9774^***^ (0.0033)	0.9659^***^ (0.0042)	0.9638^***^ (0.0079)	0.9960^**^ (0.0015)
	CW	0.9311^***^ (0.0084)	0.9614^***^ (0.0048)	0.6715^***^ (0.0338)	0.9863^***^ (0.0035)	0.9377^***^ (0.0078)	0.9636^***^ (0.0093)	0.9959^***^ (0.0014)
	SMOTE + CW	0.9203^***^ (0.0076)	0.9549^***^ (0.0045)	0.6518^***^ (0.0216)	0.9890 (0.0026)	0.9231^***^ (0.0087)	0.9632^***^ (0.0069)	0.9958^**^ (0.0012)
DMNN Intermediate fusion Concatenation	Base	0.9574^***^ (0.0059)	0.9769^***^ (0.0032)	0.7173^***^ (0.0390)	0.9701^***^ (0.0041)	0.9838^***^ (0.0045)	0.9621^***^ (0.0082)	0.9958^***^ (0.0011)
	CW	0.9362^***^ (0.0122)	0.9646^***^ (0.0070)	0.6542^***^ (0.0463)	0.9767^***^ (0.0036)	0.9529^***^ (0.0126)	0.9522^***^ (0.0097)	0.9947^***^ (0.0013)
	SMOTE + CW	0.9265^***^ (0.0098)	0.9588^***^ (0.0057)	0.6400^***^ (0.0347)	0.9811^***^ (0.0033)	0.9376^***^ (0.0101)	0.9461^***^ (0.0083)	0.9934^***^ (0.0014)
DMNN Intermediate fusion TFL	Base	0.9652^***^ (0.0053)	0.9810^***^ (0.0029)	0.7774^***^ (0.0324)	0.9787^***^ (0.0035)	0.9834^***^ (0.0044)	0.9678^***^ (0.0096)	0.9964^**^ (0.0013)
	CW	0.9457^***^ (0.0054)	0.9699^***^ (0.0031)	0.7068^***^ (0.0242)	0.9831^***^ (0.0029)	0.9571^***^ (0.0058)	0.9632^***^ (0.0091)	0.9958^***^ (0.0013)
	SMOTE + CW	0.9286^***^ (0.0112)<	0.9598^***^ (0.0065)	0.6740^***^ (0.0334)	0.9887 (0.0025)<	0.9325^***^ (0.0122)	0.9597^***^ (0.0098)	0.9950^***^ (0.0016)
DMNN Intermediate fusion MCF	Base	0.9464^***^ (0.0042)	0.9706^***^ (0.0023)	0.6770^***^ (0.0255)	0.9736^***^ (0.0034)	0.9677^***^ (0.0038)	0.9550^***^ (0.0067)	0.9948^***^ (0.0011)
	CW	0.9225^***^ (0.0073)	0.9564^***^ (0.0043)	0.6379^***^ (0.0225)	0.9836^***^ (0.0025)	0.9307^***^ (0.0082)	0.9541^***^ (0.0111)	0.9947^***^ (0.0018)
	SMOTE + CW	0.9057^***^ (0.0136)	0.9464^***^ (0.0081)	0.6060^***^ (0.0408)	0.9857^***^ (0.0035)	0.9101^***^ (0.0129)	0.9505^***^ (0.0109)	0.9943^***^ (0.0016)
DMNN Late fusion Concatenation	Base	0.9432^***^ (0.0050)	0.9691^***^ (0.0028)	0.6276^***^ (0.0310)	0.9633^***^ (0.0036)	0.9750^***^ (0.0047)	0.9414^***^ (0.0084)	0.9934^***^ (0.0012)
	CW	0.8990^***^ (0.0107)	0.9429^***^ (0.0062)	0.5476^***^ (0.0405)	0.9750^***^ (0.0049)	0.9130^***^ (0.0090)	0.9228^***^ (0.0153)	0.9912^***^ (0.0020)
	SMOTE + CW	0.9005^***^ (0.0054)	0.9434^***^ (0.0033)	0.5835^***^ (0.0181)	0.9832^***^ (0.0036)	0.9067^***^ (0.0070)	0.9381^***^ (0.0057)	0.9931^***^ (0.0009)

Table 1 shows the comparison of various prediction models via a 10-fold cross-validation, each of which is trained based on the imbalanced training dataset with or without balancing the class frequencies. We calculate means and standard deviations of the ACC, F1-score, MCC, precision, recall, AUC, and AUPRC. Boldfaced values indicate best performance result. Standard errors are given in parenthesis. As seen from Table 1, OPCNN and DMNN models overall show better results than SVM and RF for all evaluation metrics except recall. The OPCNN base model shows the highest ACC, F1-score, MCC, AUC, and AUPRC averages, which are 0.9758, 0.9868, 0.8451, 0.9824, and 0.9979, respectively. In particular, OPCNN base model significantly outperforms the other models for both F1-score and MCC that are good metrics for the imbalanced classification. Although OPCNN base model does not show the highest precision and recall averages, it still shows evenly high precision and recall averages. The DMNN base model using product operation at the early fusion step shows the second highest ACC, F1-score, and MCC averages, which are 0.9669, 0.9819, and 0.7880, respectively. If classification successes and errors must be considered together, then the MCC arises as the best choice (Luque et al., 2019). Therefore, we prefer to use MCC to assess classification performance in this study. Compared to other models, the OPCNN base model shows a significantly higher MCC average. To conclude, Table 1 shows that OPCNN base model is the best model for predicting successes and failures of clinical trials.

**FIGURE 3:**
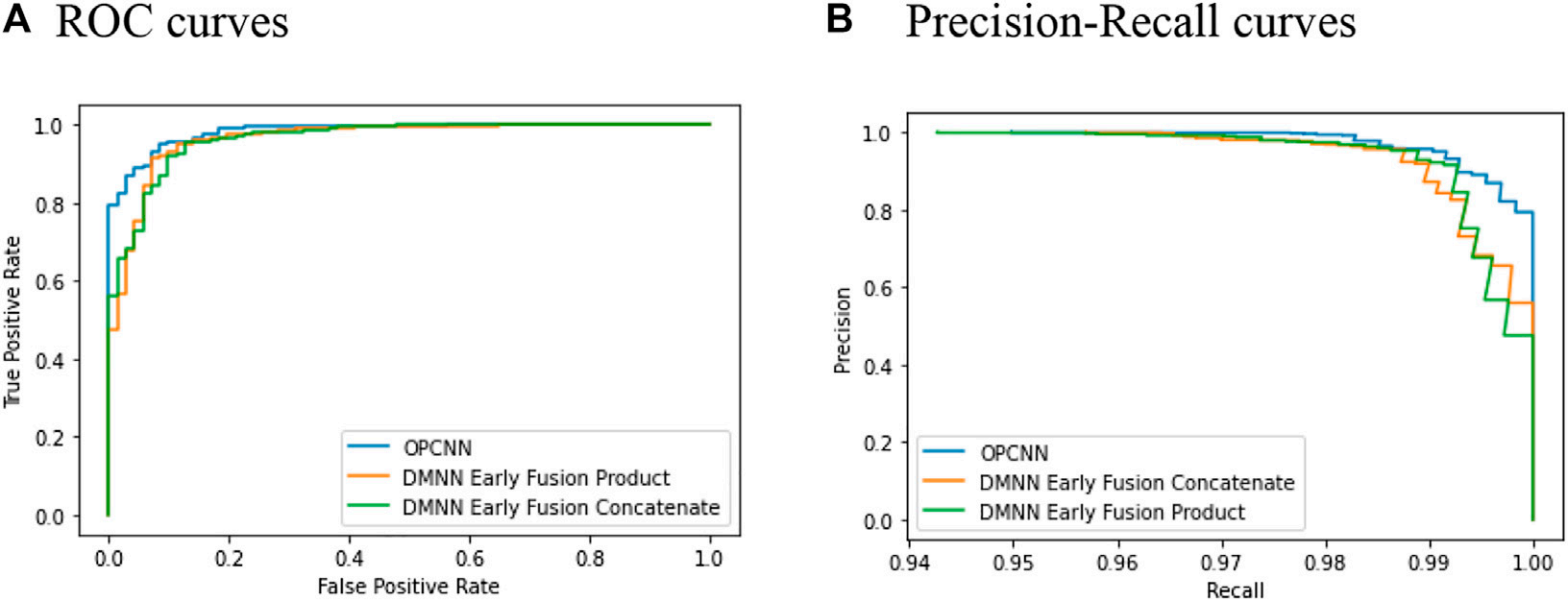
ROC and precision–recall curves for 10-fold cross-validation. **(A)** ROC curves **(B)** Precision–recall curves.

Plotting ROC and precision–recall curves is a popular way for discriminatory accuracy visualization of the binary classification models. Figure 3 shows the graph of ROC curves and precision–recall curves for three best-performing models in terms of AUC and AUPRC, respectively. Since we replicate the cross-validation 20 times for each model, we here show curves only for one replication. Figure 3 shows that the OPCNN base model is a better classifier. By the way, Table 1 illustrates that AUC averages of these three models differ significantly but AUPRC averages of these three models do not differ significantly.

## Conclusion

In this study, to develop the prediction model of the outcomes of clinical trials of drug candidates, we proposed OPCNN model that employs the augmented outer product to join effectively chemical features of drugs and target-based features. The proposed OPCNN model was evaluated via 10-fold cross-validation techniques on dataset used in Gayvert et al. (Gayvert et al., 2016), which consists of 757 approved drugs for positive class and 71 failed drugs for negative class. We observed that the OPCNN base model shows the highest averages of ACC, F1-score, MCC, AUC, and AUPRC. In particular, it is noteworthy that the OPCNN base model showed the highest averages of F1-score, MCC, and AUPRC, which are more reliable metrics for the imbalanced classification. The two-sided t-test showed that F1-score and MCC averages of OPCNN base model are significantly higher than those of the other models. The OPCNN base model also showed evenly high precision and recall averages, even though this model did not show the highest precision and recall averages. The graph of ROC curves and precision–recall curves also illustrate that the OPCNN base model is a better classifier.

Although we did not report the experimental results, we also conducted experiments on ensemble models based on RFs, extra trees, and weighted least squares SVM. In addition, we performed experiments on a DMNN using a one-dimensional CNN for each individual modality. OPCNN and DMNN models aforementioned performed much better than those of ensemble models for all of five evaluation metrics. The purpose of this study is to develop an efficient predictive model based on the dataset used in Gayvert et al. (Gayvert et al., 2016). The key idea underlying OPCNN is to integrate two modalities using the augmented outer product and to apply CNN to the resulting matrix. We think this idea can be effectively applied to other tasks based on bimodal data and can be extended to multimodal data. The OPCNN model can be further improved by adjusting the architecture of CNN according to the data structure.

A critical issue is that the dataset does not have enough samples for the use of deep learning and particularly has only 71 samples for failure data. Therefore, OPCNN and DMNN could overfit the data since these complex models are likely to detect subtle patterns in the data. Obviously, these patterns will not generalize to new instances. Therefore, we need to apply our OPCNN to a larger dataset and check its efficacy. Furthermore, we need to carefully argue that our OPCNN is an effective approach for predicting successes and failures of clinical trials and can be quite helpful in drug development process.

## Data Availability

The dataset and source code for this paper can be downloaded from the Github repository at https://github.com/sawoo9410/Clinical-Trials-with-OPCNN.
